# Predicting Late Adolescent Anxiety From Early Adolescent Environmental Stress Exposure: Cognitive Control as Mediator

**DOI:** 10.3389/fpsyg.2020.01838

**Published:** 2020-08-11

**Authors:** Nancy Tsai, Susanne M. Jaeggi, Jacquelynne S. Eccles, Olivia E. Atherton, Richard W. Robins

**Affiliations:** ^1^McGovern Institute for Brain Research, Massachusetts Institute of Technology, Cambridge, MA, United States; ^2^School of Education, University of California, Irvine, Irvine, CA, United States; ^3^Department of Psychology, University of California, Davis, Davis, CA, United States

**Keywords:** cognitive control, executive function, self-regulation, mental health, stress

## Abstract

Early exposure to stressful life events is associated with greater risk of chronic diseases and mental health problems, including anxiety. However, there is significant variation in how individuals respond to environmental adversity, perhaps due to individual differences in processing and regulating emotional information. Differences in cognitive control – processes necessary for implementing goal directed behavior – have been linked to both stress exposure and anxiety, but the directionality of these links is unclear. The present study investigated the longitudinal pathway of environmental stress exposure during early adolescence on later adolescent anxiety, and the possible mediating mechanism of cognitive control. Participants were 674 Mexican-origin adolescents (mean_age_ = 10.8 years, 50% male) enrolled in the California Families Project, an ongoing longitudinal study of Mexican-origin families. In the current analysis, we examined self-reports of environmental stressors at age 14 (Time 1), cognitive control at age 16 (Time 2), and anxiety at age 18 (Time 3). Structural equation modeling revealed that environmental stressors (Time 1) had both direct and indirect effects on later anxiety (Time 3) through their effects on cognitive control (Time 2), even when accounting for prior levels of anxiety (Time 2). Cognitive control accounted for 18% of the association between environmental stressors and adolescent anxiety: an increase in stressors decreased cognitive control (β = −0.20, *p* < 0.001), however, cognitive control buffers against anxiety (β = −0.10, *p* = 0.004). These findings deepen our understanding of the mechanisms underlying the development of anxiety and highlight the importance of cognitive control as a potential protective factor.

## Introduction

Exposure to stressful life events is associated with greater risk of developing chronic diseases and mental health disorders, including anxiety – the most prevalent psychiatric disorder experienced by youth (Pérez-Edgar and Fox, 2005; Pine, 2007; Rapee et al., 2009). However, there is significant variation in how individuals respond to environmental adversity, and those individual differences might be related to the processing and regulation of information. These cognitive control processes necessary for implementing goal-directed behavior – an umbrella term for a collection of related yet distinct processes that are also labeled effortful control, executive function, and self-regulation, depending on the field of study (Zhou et al., 2012) – have been significantly associated with anxiety, as well as a range of other psychiatric disorders such as depression and substance abuse (e.g., Baler and Volkow, 2006; Hirsch and Mathews, 2012; Zainal and Newman, 2018). Cognitive models of generalized anxiety disorder have converged on cognitive control as a potential mechanism of psychopathology (Joormann, 2006; Joorman et al., 2009; Hirsch and Mathews, 2012). For example, the Attentional Control Theory (Eysenck and Derakshan, 2011) and the Processing Efficiency Theory (Eysenck and Calvo, 1992) were put forth to explain the reallocation of cognitive resources when processes such as inhibition, stress, and negative thoughts co-occur. These theories postulate that compromised cognitive control is linked to excessive and uncontrollable worry, a core symptom of anxiety. Indeed, cross-sectional studies have found an association between a range of cognitive control functions and anxiety disorders (e.g., Toren et al., 2000; Muris and Ollendick, 2005; Fujii et al., 2013), as well as the degree of cognitive control impairment being commensurate with the severity of anxiety among patients with generalized anxiety disorder (Hallion et al., 2017). The few studies examining longitudinal associations have found that executive function is related to anxiety problems in an adolescent population two years later (Han et al., 2016) and in adults nine years later (Zainal and Newman, 2018). The scarcity of studies examining longitudinal associations between of cognitive control and anxiety begs the question of directionality and whether cognitive control is an underlying mechanism that might mediate the effect of stress exposure on the development of anxiety.

Stress and cognitive control are processed by closely related neural systems (e.g., Herman et al., 2005), leading some researchers to speculate that stress exposure during childhood and adolescence, sensitive periods of neurocognitive development, can compromise the development of the neural regions that underlie the development of cognitive control (Shonkoff and Phillips, 2000; Lupien et al., 2009). For example, a longitudinal study of infants raised in a predominantly low-income environment found that the chronic physical and psychosocial stress exposure associated with poverty predicted later executive function in pre-kindergarten (Berry et al., 2012). In their longitudinal study examining childhood poverty (age 9) and later adult emotion regulation (age 24), Kim et al. (2013) found that cumulative chronic stress mediated the relationship, mimicking findings highlighting the mediating role of stress between childhood poverty and later cognitive control (e.g., Evans and Schamberg, 2009; Blair et al., 2011; Evans and Fuller-Rowell, 2013; Kim et al., 2018). These findings underscore the link between early stress exposure to later diminished executive function abilities (Gunnar et al., 2009; Blair, 2010; Ursache et al., 2013), but whether these relationships together explain anxiety outcomes remains unclear.

Although the aforementioned links all strongly suggest a mechanism by which early experiences of stress contribute to anxiety outcomes, few published studies to date have explicitly examined the relationship between stress exposure, cognitive control, and anxiety together. In a recent study, Huh et al. (2017) found mediating effects of emotion regulation (i.e., cognitive control in emotionally salient contexts) on the relationship between acute childhood stressors and adult anxiety symptoms in a clinical population using a cross-sectional design. Similarly, Affrunti and Woodruff-Borden (2015) found that executive functions mediated the relationship between fear perception and anxiety in 7- to 10-year-old children. With a short-term longitudinal design, Gulley et al. (2016) examined the interaction of effortful control and stressors on the development of anxiety over a 3-month period finding that, at low levels of stress, high level of effortful control protected against the development of anxious symptoms. With little to almost no prospective studies to draw from, some have speculated that adolescents with more effective cognitive control abilities are better able to process negative emotional information, which in turn lowers their risk for psychopathology (Martel et al., 2007; Micco et al., 2009). Similarly, Nigg (2006) theorizes that anxiety arises from experiences of both negative affect and impaired cognitive control. That is, greater exposure to stressors paired with lower levels of cognitive control may contribute to increased anxiety and depression (Anthony et al., 2002; Eisenberg et al., 2005). However, no studies to date have tested these theories by examining the longitudinal relations between early environmental stress exposure, cognitive control, and later anxiety, and thus, the directionality of these relationships remains unclear and beckons the need for further research.

In the present study, we conducted a prospective mediation analysis to evaluate the effect of environmental stress exposure during early adolescence on late adolescent anxiety and examine the possible mediating mechanism of cognitive control. Given previous findings, we hypothesize that:

(1) Increased stress exposure is associated with higher levels of anxiety.
(2) This relation between stress exposure and anxiety is partially mediated by cognitive control, with increased stress exposure leading to impaired cognitive control, whereas cognitive control in turn buffers against the development of anxiety.

Previous research examining the association between environmental stress exposure and anxiety has often operationalized environmental stress as poverty, leaving a vast range of other possible environmental stressors overlooked and/or under examined. Thus, the present study uses data from a sample of Mexican-origin youth in the United States who face unique challenges and may experience greater exposure to adversity ranging from fewer material and emotional resources to increased exposure to discrimination and neighborhood violence, and more chaotic and less stable home environments (Evans and Kantrowitz, 2002; Evans and Kim, 2010), experiences that can cause chronic stress beyond those of financial origins. Moreover, data from this sample of youth provide an opportunity to examine changes in cognition and anxiety in the context of adolescence – a unique developmental time period marked by notable neurocognitive changes and heightened prevalence of stress-related psychological disorders (Merikangas et al., 2010; Romeo, 2017). Thus, it is critical for research to elucidate the potential risk factors that lead to the development of these disorders during this period of enhanced vulnerability.

## Materials and Methods

### Participants

Participants were 674 Mexican-origin adolescents (mean_age_ = 10.8 years, 50% male) enrolled in the California Families Project, an ongoing 12-year longitudinal study of Mexican-origin families (for additional details about the study, see Martin et al., 2019; Atherton et al., 2020). Of the 674 participants, 551 participants had longitudinal data for all our variables of interest at ages 14 (Time 1), 16 years (Time 2), and 18 years (Time 3) and were included in the analysis. Potential participants were drawn at random from rosters of students from the Sacramento and Woodland, CA, school districts. To be eligible for participation in this study, the focal child had to be in the fifth grade, of Mexican origin, and living with his/her biological mother; 72.6% of the eligible families consented to participate in the study, which was granted approval by the Institutional Review Board of University of California, Davis.

### Measures

#### Environmental Stressors

We measured environmental stress exposure using a composite of three separate scales that were all administered at age 14: neighborhood criminal events, neighborhood quality dissatisfaction, and adolescent reports of discrimination experiences. Similar to other composite measures such as socioeconomic status, which is often defined as a composite of occupation, education, and income, our measure of environmental stress exposure is a formative construct: the events are largely independent of each other but collectively contribute to the construct (see Edwards and Bagozzi, 2000). Therefore, environmental stress exposure was calculated by summing the average scores of all three risk factors. Additive indices of cumulative stress exposure are robust and consistently predict mental health outcomes better than indices of singular stress exposure or alternative multiple stress exposure metrics (Evans et al., 2013; Kim et al., 2013; Evans and Cassells, 2014) and have been established as a reasonable method in capturing the confluence of physical and psychosocial challenges associated with adolescent adversity (Evans and English, 2002).

#### Neighborhood Criminal Events Scale

The adolescent reported on neighborhood-level violence using the Neighborhood Criminal Events Scale, which consists of 10 items. These items assess the extent to which there is violence and disorder in the neighborhood (Aneshensel and Sucoff, 1996; Bowen and Chapman, 1996; Sampson et al., 1997; Cutrona et al., 2000; Ross and Jang, 2000). The scale includes items, such as “How often did [violent crimes including stabbings, shootings, and violent assaults] happen in your neighborhood in the past year?” and “How often did [kids sell illegal drugs] in your neighborhood in the past year?” Ratings were made on a four-point scale ranging from 1 (almost never or never) to 4 (almost always to always). Higher scores indicated greater exposure to crime. The scale demonstrated good internal reliability (α = 0.88).

#### Neighborhood Quality Dissatisfaction

The adolescent reported on his/her personal evaluation of attractiveness of the neighborhood using an abbreviated version of Neighborhood Quality Evaluation Scale (Roosa et al., 2005), which consists of six items. A typical item is “Your neighborhood is clean and attractive” and “Overall, you are satisfied with your neighborhood.” Ratings were made on a four-point scale ranging from 1 (not at all true) to 4 (very true). Higher scores indicated higher perceptions of neighborhood quality. The average score was then reversed to reflect negative neighborhood quality, with higher scores indicating poorer perceptions of neighborhood quality, which was then used as part of the cumulative stressor score. This scale demonstrated excellent internal reliability (α = 0.93).

#### Perceived Ethnic Discrimination

The adolescent reported his/her perceived personal experiences with ethnic discrimination using four items, which were adapted for use in the La Familia Project (Johnston and Delgado, 2004) from questions on the Racism in the Workplace Scale (Hughes and Dodge, 1997) and Schedule of Sexist Events (Klonoff and Landrine, 1995). Sample items include “You have heard your teachers at school making jokes or saying bad things about [Mexicans/Mexican–Americans]” and “Teachers think kids who speak Spanish don’t do as well at school.” Ratings were made on a four-point Likert scale, ranging from 1 (almost never or never) to 4 (almost always or always). Higher scores indicated greater experiences of discrimination. The scale demonstrated adequate internal reliability (α = 0.68).

#### Cognitive Control

Adolescents completed the effortful control scale (16 items) from the short form of the Early Adolescent Temperament Questionnaire – Revised when the adolescent was 16 years old (EATQ-R; Ellis and Rothbart, 2001). The 16-item EATQ-R scale assesses various aspects of cognitive control including the capacity to perform an action when there is a strong tendency to avoid it, the capacity to focus and shift attention when desired, and the capacity to suppress and regulate dominant impulses. This scale includes items such as “When someone tells you to stop doing something, it is easy for you to stop.” and “You pay close attention when someone tells you how to do something.” Ratings were made on a four-point scale ranging from 1 (not at all true of you) to 4 (very true of you). Higher scores indicated greater cognitive control. The full scale demonstrated adequate reliability (α = 0.65). Cognitive control assessed at age 16 (Time 2) was included as our mediator of interest.

#### Anxiety

Anxiety was assessed using the Mini-Mood and Anxiety Symptom Questionnaire (Casillas and Clark, 2000). For our measure of anxiety, we composited the anxiety (three items; “How much have you felt keyed up or on edge”) and anxious arousal (10 items; “Have you had trouble swallowing”) items into an overall anxiety scale. Participants rated how much they “felt or experienced” each symptom “during the past week” using a four-point scale ranging from 1 (not at all) to 4 (very much). Higher scores indicated more anxiety. The scale demonstrated good reliability (α = 0.87). Anxiety assessed at age 16 (Time 2) was included as a covariate given that our outcome of interest was anxiety at age 18 (Time 3).

### Analytical Approach

Our prospective mediation analysis was framed around three time points (Time 1, 2, 3) in order to capture a full prospective mediation model. Several considerations informed the development of our analytical model: (1) the temporal sequence of variables required in a mediation model; (2) the need to account for the stability of anxiety over time, to ensure that the effects of environmental stress and cognitive control are, in fact, prospectively predicting anxiety (and not just due to the fact that anxiety symptoms are stable across adolescence); and (3) the equivalent distance of time between measurements. Our final model was determined by these constraints and captures the development of these constructs during the peak of adolescence. Thus, we examined self-reports of environmental stressors at age 14 (Time 1), cognitive control at age 16 (Time 2), and anxiety at age 18 (Time 3), while including anxiety at age 16 as a covariate^1^.

To address our research questions, we conducted a prospective mediation analysis using SEM in Stata Version 13 (StataCorp, 2013). Bootstrapping procedures in SEM were used to test the significance of the mediation effects of cognitive control. In this study, 200 bootstrapping samples were generated from the original data set by random sampling to determine indirect effects of mediating variables and analyze the corresponding confidence intervals. This statistical approach is considered to be a robust method of analyzing indirect effects (Hayes, 2009).

## Results

Descriptive statistics, correlations, and α reliability estimates for all study variables were calculated prior to addressing our research questions and are displayed in Table 1. The hypothesized structural model comprised three observed variables: environmental stressors at age 14 (Time 1), cognitive control at age 16 (Time 2), and anxiety at age 18 (Time 3). In addition, we included anxiety at age 16 (Time 2) as a predictor of anxiety at age 18 (Time 3) in order to account for the fact that anxiety symptoms are likely stable over time and allow us to draw stronger inferences about prospective effects.

**TABLE 1:** Mean, Standard Deviations (SD), and pairwise correlations among study measures.

Measure	Time at measurement	Age atmeasurement	*Mean*	*SD*	1	2	3
1. Environmental stressors	T1	14	1.67	0.44			
2. Cognitive control	T2	16	2.93	0.37	−0.19**		
3. Anxiety at Age 16	T2	16	1.3	0.3	0.18**	−0.33**	
4. Anxiety at age 18	T3	18	1.22	0.26	0.17**	−0.21**	0.35**

The presentation of the pairwise correlations focus on the measures at timepoints relevant to the analysis. Correlation is significant at **p < 0.001.

**FIGURE 1:**
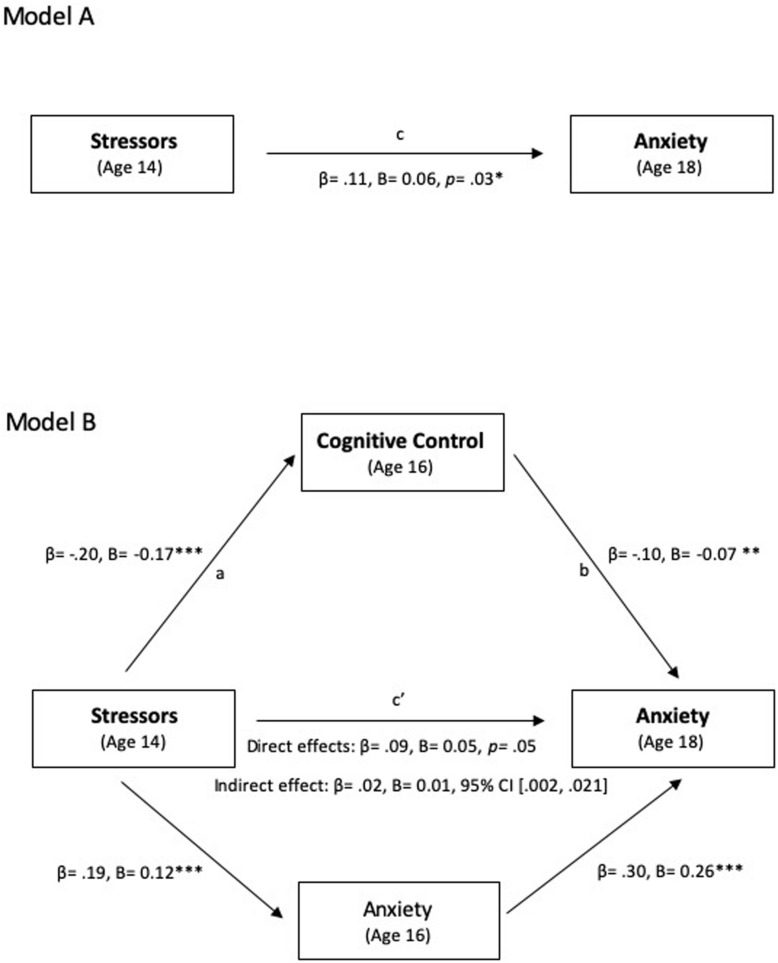
Path model showing the effect of cumulative environmental stressors on anxiety mediated by cognitive control. Total effects model **(A)** and indirect effects model **(B)** demonstrate the cognitive control at age 16 partially mediated the relation between environmental stressors at age 14 and anxiety at age 18. Anxiety at age 16 is included as a covariale. Residual variances of cognitive control and anxiety at age 16 are correlated to account for association due to unmeasured common causes (*r* = −0.03). Both standardized and unstandardized coefficients are shown. *p* < 0.05, ^∗∗^*p <* 0.01, ^∗∗∗^*p* < 0.001.

We hypothesized that individuals with higher levels of environmental stress exposure would later report higher levels of anxiety, as compared with peers with lower levels of environmental stress exposure (Hypothesis 1). Furthermore, we predicted that this effect would be mediated by cognitive control (Hypothesis 2). Indeed, structural equation modeling revealed that cumulative environmental stressors at age 14 had both direct (path c′, Figure 1B) and indirect (paths a and b, Figure 1B) effects on later anxiety at age 18 through their effects on cognitive control at age 16 even when previously reported anxiety at age 16 was included as a covariate. Figure 1 shows the results of a test of the full model, including the total (Figure 1A) and indirect effects (Figure 1B) among cumulative environmental stressors, cognitive control, and anxiety.

Consistent with Hypothesis 1, our results show that adolescents who report higher levels of cumulative environmental stressors at age 14 later reported greater anxiety at age 18 (c; β = 0.11, *B* = 0.06, *z* = 2.68, *p* = 0.02). Consistent with Hypothesis 2, which predicts that cognitive control would mediate the relation between cumulative environmental stressors and early adulthood anxiety, our results demonstrate a significant effect of environmental stressors on cognitive control (a; β = −0.20, *B* = −0.17, *z* = −4.52, *p* < 0.001), of cognitive control on anxiety (b; β = −0.10, *B* = −0.07, *z* = −2.91, *p* = 0.004), and of environmental stressors on anxiety (c′; β = 0.09, *B* = 0.05, *z* = 2.00, *p* = 0.05). These effects remained statistically significant even while controlling for anxiety at age 16, which suggests that cognitive control as a mediator is prospectively predicting anxiety at age 18 over and above prior levels of anxiety. That is, those reporting higher levels of environmental stressors tended to have lower cognitive control. Higher cognitive control, in turn, was associated with lower levels of late adolescent anxiety. The bootstrapped unstandardized indirect effect was *B* = 0.012, confidence interval [0.002, 0.02], and thus, the indirect effect was statistically significant. As a partial mediator, cognitive control accounted for 18% of the association between environmental cumulative stressors and adolescent anxiety (indirect effect/total effect): individuals reporting higher levels of cumulative environmental stress exposure tended to have decreased cognitive control (β = −0.20, *B* = −0.17, *p* < 0.001), cognitive control in turn was associated with decreased anxiety (β = −0.10, *B* = −0.07, *p* = 0.004). Taken together, these findings are consistent with the hypothesis that cumulative environmental stress exposure is associated with later anxiety at least in part because stress exposure impairs cognitive control, a critical factor in buffering against the development of anxiety.

## Discussion

The purpose of the current study was to investigate the potential meditational role of cognitive control in the longitudinal relation between cumulative environmental stress exposure and the development of late adolescent anxiety. Given previous research, we tested the hypotheses that (1) increased stress exposure would be associated with higher levels of anxiety, and (2) this association would be partially mediated by cognitive control, with increased stress exposure being associated with impaired cognitive control, which in turn is linked to increased anxiety.

In line with our first hypothesis, our findings revealed a statistically significant positive association between early adolescent cumulative environmental stress exposure and later adolescent anxiety, albeit with β = 0.11, the effect is considered small (small = 2%, medium = 15%, and large = 25%; Lachowicz et al., 2018). This is consistent with a large body of research demonstrating a link between early exposure to adverse experiences and a range of later physical and mental health outcomes (see Nusslock and Miller, 2016, for review). However, our findings point to the importance of examining stress exposure from different sources. Previous studies have examined child maltreatment, poverty, family instability, socioeconomic status, and trauma to operationalize stress and adversity. In our unique sample, we touched upon a small fraction of the breadth of stressors one may be exposed to during development. We included reports of discrimination, exposure to criminal activity, and neighborhood quality in our measure of cumulative environmental stress. Sources of stress are wide ranging – from health inequalities to experiences of racism and discrimination – therefore, we urge these diverse experiences of stress to be reflected in future research and to be considered for their potential cumulative effects.

In line with our second hypothesis, we found the aforementioned relationship was partially explained by adolescent cognitive control. Specifically, our findings demonstrated that cognitive control mediated the relation between cumulative environmental stress exposure at age 14 and anxiety at age 18: those with greater exposure to environmental stressors tended to then have lower cognitive control (medium effect; β = −0.20), but higher cognitive control, in turn, was associated with lower levels of late adolescent anxiety (small effect; β = −0.10). In fact, even after controlling for anxiety at age 16, our tested model demonstrates that cognitive control accounts for 18% of the total effect between environmental stress exposure at age 14 and anxiety at age 18, which indicates a medium proportion of explained variance (Lachowicz et al., 2018). As the first study to examine the three constructs in a prospective, longitudinal manner, our results converge with evidence from developmental psychology, public health, and neuroscience to chronicle the role of social systems in shaping the development of our mental and emotional health. More importantly, our findings uniquely identify cognitive control as an underlying mechanism, a protective factor that is both vulnerable to the influences of environmental stress yet potentially buffers against these deleterious effects on anxiety outcomes. Thus, efforts to mitigate mental health outcomes for youth ought to consider the role and malleability of cognitive control. Although results from cognitive control interventions are mixed (see Au et al., 2015 for meta-analysis), a growing number of interventions studies have shown some promise in improving mental health outcomes (Owens et al., 2013; Koster et al., 2017; Jopling et al., 2020). The prospect of optimizing this function is critical in promoting resilience, particularly during adolescence, a unique period of neurocognitive development and enhanced vulnerability.

It is important to note that despite the fact that the above relations were statistically significant, their β values ranged from small to medium. Specifically, the relation between cumulative environmental stress at age 14 and later anxiety at age 18 may be meaningful but smaller than expected given the findings from previous literature. It is important to note, however, that previous findings were either cross-sectional in nature and/or examined only two constructs, which may magnify the strength of the relationships. A longitudinal study examining poverty, chronic stress, and later cognitive control – as indexed by neural activity – reported similar β values for poverty and cognitive control ranging between 0.03 and 0.05, and for chronic stress and cognitive control ranging between −0.13 and −0.14 (Kim et al., 2013), which closely mirrors our findings for the direct effects of path a and c′ (Figure 1). As such, modest values may reflect the challenges in isolating causal mechanisms that are inherent to longitudinal work, where the dynamic relationship of variables gets diluted over time as other factors come into play. For example, our findings demonstrate that concurrent measurement of cognitive control and anxiety at age 16 leads to a stronger relationship (*r* = −0.33, *p* < 0.001) than cognitive control at age 16 and anxiety at age 18 (*r* = −0.21, *p* < 0.001). Note, however, that the indirect effect of cognitive control accounted for 18% of the total relationship, despite the weakened association over the 2 years and controlling for anxiety at age 16.

The present results should be considered in light of a few limitations. First, our measure of environmental stressors aimed to encapsulate the cumulative effects of stress through measures of environmental adversity. The range of measures included – from perceived discrimination to neighborhood quality – resulted in modest correlation between measures. However, this modest correlation may reflect the methodological issues of assessing environmental stress. Measurement has taken on a variety of forms in attempt to capture the broad range of physical to psychosocial sources (see Evans and English, 2002 for review). One promising approach indexes environmental stress exposure as a cumulative construct in attempt to capture the confluence of multiple external demands that may lead to suboptimal outcomes for youth. Literature on chronic stress shows that the *quantity* of risk factors encountered, as captured by a cumulative index, and not the particular *type* that seems to better predict outcomes (Kraemer et al., 2005; Sameroff, 2006; Evans and Cassells, 2014). With our cumulative score from three questionnaires, the self-reported levels of chronic stress were low in our sample (mean = 1.67, range = 0–4), which could reflect measurement issues and/or the possibility that our sample was not exposed to high levels of environmental stress. Future work would benefit from including a greater breadth of measures for a more robust index.

Second, our measure of cognitive control relied on self-report. In a meta-analysis of 282 studies of self-control, correlations within and across types of self-control measures were weak (Duckworth and Kern, 2011). Future work including some combination of behavioral, observational, and self-report may improve measurement validity. Lastly, the direction of the relationship between cognitive control and anxiety is arguable. That is, there is literature indicating impaired cognitive control *causes* anxiety (Abravanel and Sinha, 2015), anxiety *causes* impaired cognitive control (Edwards et al., 2016), or that cognitive control moderates the relationship between stress and adversity on poor mental health outcomes (Extremera and Rey, 2015). Although correlational in nature, our novel findings hint at the first causal effect – that is, greater cognitive control is associated with later decreased anxiety – but all three effects have not been adequately examined together (as competing or complementary processes) in a longitudinal context.

The current study set out to synthesize findings from stress, cognition, and mental health literature and test previously untested theories on the directionality of these relationships during adolescence. As the first prospective longitudinal study in this area, our results deepen our understanding of the mechanism underlying early stress exposure and the development of anxiety during a developmentally sensitive period. More importantly, our findings underscore the importance of preserving cognitive control as a means of combating mental health disorders and as a possible protective factor in promoting resilience.

## Data Availability Statement

The datasets generated for this study will not be made publicly available due to confidentiality reasons. Data is available upon application through administrators of the California Families Project.

## Ethics Statement

The studies involving human participants were reviewed and approved by the Institutional Review Board of University of California, Davis. Written informed consent to participate in this study was provided by the participants’ legal guardian/next of kin.

## Author Contributions

NT, SJ, JE, and RR contributed to the theoretical development of the study. OA supplied resources needed for study analysis. NT performed the data analysis and interpretation under the supervision of SJ, JE, and RR. NT drafted the manuscript. SJ, JE, RR, and OA provided revisions. All authors approved the final version of the manuscript for submission.

## Conflict of Interest

The authors declare that the research was conducted in the absence of any commercial or financial relationships that could be construed as a potential conflict of interest.

## Footnotes

1 Sex was also examined as a second covariate but did not change any of the findings (i.e., all significant effects remained significant), and thus was omitted from the final model presented here.
